# Retraction Note: Dietary garcinol supplementation improves diarrhea and intestinal barrier function associated with its modulation of gut microbiota in weaned piglets

**DOI:** 10.1186/s40104-024-01134-0

**Published:** 2024-12-02

**Authors:** Tongxin Wang, Weilei Yao, Juan Li, Yafei Shao, Qiongyu He, Jun Xia, Feiruo Huang

**Affiliations:** Department of Animal Nutrition and Feed Science, College of Animal Science and Technology, Huazhong Agricultural University, Wuhan, 430070 China

**Retraction Note** **:** **Journal of Animal Science and Biotechnology 11, 12 (2020)**

**https://doi.org/10.1186/s40104-020-0426-6**

The Editor-in-Chief has retracted this article at the request of the Huazhong Agricultural University. An institutional investigation determined that there are concerns regarding the reliability of some of the data presented in this work, specifically, Figures 4 and 5, and Tables 4, 6, 7, and 11. These concerns call into question the integrity of the data and the article's overall scientific soundness. The Editor-in-Chief therefore no longer has confidence in the research presented in this work.

The authors have not replied to correspondence from the Publisher.

